# Scoping review meets expert interviews: Key issues of multimodal programs for workplace health promotion in long-term care facilities – *"We can’t just run a standard program"*

**DOI:** 10.34172/hpp.42899

**Published:** 2024-10-31

**Authors:** Judith Czakert, Anne Schirmaier, Sarah B. Blakeslee, Wiebke Stritter, Anna K. Koch, Christian Kessler, Georg Seifert

**Affiliations:** ^1^Charité - Universitätsmedizin Berlin, corporate member of Freie Universität Berlin and Humbold-Universität zu Berlin, Charité Competence Center for Traditional and Integrative Medicine (CCCTIM), Berlin, Germany; ^2^Charité - Universitätsmedizin Berlin, corporate member of Freie Universität Berlin and Humbold-Universität zu Berlin, Institute of Social Medicine, Epidemiology and Health Economy, Berlin, Germany; ^3^Department of Internal and Nature-Based Therapies, Immanuel Hospital Berlin, Berlin, Germany; ^4^Clinical Hospital, Faculty of Medicine, University of São Paulo, São Paulo, Brazil

**Keywords:** Health promotion, Integrative medicine, Interview, Long-term care, Nursing care, Qualitative research, Review

## Abstract

**Background:**

Long-term care facility employees’ workload escalation intensifies negative risk for (nursing) staff health, residents, and the economy. Workplace health promotion (WHP) has emerged as a vital approach with positive impacts on employee well-being. This Scoping Review focuses on multimodal WHP programs in long-term care facilities, emphasizing barriers, facilitators, and the integration of complementary and integrative medicine (CIM).

**Methods:**

Following the PRISMA-ScR guidelines, a systematic search strategy from February to April 2023 in Medline (PubMed), EMBASE (Ovid), and CINAHL (EBSCOhost) yielded 506 hits. Findings were enriched through semi-structured expert interviews. All data were analyzed with a deductive-inductive qualitative content analysis.

**Results:**

Eleven publications met inclusion criteria and eight experts were interviewed based on key topics of the included publications. The integration of the results showed that primary obstacles highlight structural challenges (time, finances, hierarchies) and team dynamics (lack of support, communication issues, low motivation), while effective communication, support from the management, and participatory engagement enhance program success. CIM approaches are not explicitly referenced as such, despite widespread use.

**Conclusion:**

An exclusive CIM-focus, with multimodal WHP programs for long-term care facilities is missing and should highlight the necessity of a multimethod approach intervention. While the need for further research about the specific topic of multimodal, CIM-based WHO programs in long-term care facilities – including cross-cultural and international comparisons – is apparent, an appropriate evaluation of complex interventions is challenging given the nature of multimodal WHP programs. A multi-method approach is therefore recommended as standard for further research in this area.

## Introduction

 The number of people in need of full inpatient care living in Germany rose to 24.5% between 2005 and 2019, i.e. to a total of 818 000 people in need of care.^1^ A further increase can be expected in the upcoming decades.^2^ These increasing numbers are likely to exacerbate structural challenges, mainly characterized by staff shortages and understaffing,^3^ and further intensify the pressure on professional caregivers. That might be expressed in both psychological (e.g., time pressure, work interruptions, high work intensity, work compression) and physical stressors (e.g., heavy lifting/carrying, forced postures, prolonged standing work).^4^ In long-term care facilities,^5^ the tendency toward above-average mental and physical stress on caregivers due to staff shortages, time pressure, high workloads, work organization and physical demands is particularly apparent.^6-8^ This also includes experiences of violence and a high work-life conflict.^9^ Potential consequences for the professional caregivers in long-term care facilities such as burnout, emotional exhaustion, depression, reduced working performance, dissatisfaction with work, etc. can be reflected also in the decreasing quality of care provided to the residents.^4,10-13^ Further secondary effects are seen in economic burden due to accompanied sick leave which is particularly high in long-term care facilities.^6^ Evidence indicates that interventions to reduce stress among health care workers can be economically profitable for employers.^14,15^ Also, interventions to improve healthy lifestyles, for example regarding healthy nutrition, physical activities and health education, show positive effects on caregivers’ general health and well-being,^11,12,16^ and have the potential to be economically profitable in the long run. Correspondingly, workplace health promotion (WHP) is seen as an organizational investment in the future.^17^

 One strategy to deal with the challenges outlined above is to strengthen the WHP in long-term care facilities. Multiple reviews have summarized the state of research regarding WHP for nurses and professional caregivers.^15,18-21^ However, to our current knowledge, only one review has focused on WHP within the specific setting of long-term care facilities.^22^ The results suggest that multicomponent interventions may have a positive impact on nurses’ physical and mental health. The potential of individualized, multimodal WHP programs for nurses is also highlighted elsewhere.^23^ The high acceptance of complementary and integrative medicine (CIM) approaches among both staff and residents of long-term care facilities shows another promising potential for WHP programs. There is also evidence that WHP programs with integrated CIM approaches can improve the relationship between carers and residents.^24^ CIM techniques – which include evidence-based natural therapies such as mind-body medicine, aromatherapy, yoga, and traditional medicine systems – are often inherently resource-oriented and health-promoting by following a salutogenetic, holistic approach.^25^ The procedures are individually adaptable to the needs, and conditions of both individuals, and groups of people and can thus be profitably established in everyday life outside the work context. Accordingly, a number of reviews of WHP for health care professionals have focused on interventions that can be classified within the spectrum of CIM, such as Mindfulness Based Stress Reduction (MBSR),^18,19^ and Tai Chi.^20^ Others mention approaches that can be associated with CIM, in part without explicitly referring to this discourse.^12,15,21,26^ Against this background, there is some evidence that a multimodal WHP program, composed of CIM approaches, could lead to a particularly good and sustainable acceptance among health care staff. Despite the indication of the ability of WHP strategies to strengthen the health of professional caregivers in long-term care facilities and numerous developed and implemented approaches, a sustainable adoption over time is lacking. The reasons described are, for instance, due to *“workplace culture, occupational factors such as job stress and rotating shift and intrinsic factors (e.g., absence of motivation and financial constraints)”.*^27^

###  Objective and research questions

 In order to build on existing evidence and experience for the future development of WHP programs for long-term nursing facilities, a scoping review – extended, supported and deepened by an explanatory sequential mixed-method approach with empirical data from expert interviews – was conducted.^28^

 The objective of the review is to identify knowledge and experience about multimodal WHP programs in long-term care facilities, and barriers/ challenges regarding a sustainable implementation. Based on this, a framework will summarize the most important aspects of designing, implementing, and improving such programs. Specifically, the framework is meant to provide orientation for the development of a multimodal WHP program for professional caregivers/ nurses with CIM approaches in German long-term care facilities as part of an upcoming project. The participation-based project involves conceptualization, implementation, and evaluation of a multimodal, CIM-based WHP program for employees of long-term care facilities. By integrating experiences of experts from the field into the scoping review (SR), specific conditions, challenges, resources, and lessons learned from Germany are considered.

 The underlying research question is:What are the key factors and characteristics concerning multimodal WHP programs in long-term care facilities?

 A number of sub-questions that are adapted to the objective of the review further differentiate the overall question:

Which barriers and challenges are described?
Which implications for future development of multimodal WHP programs can be derived from the results?
(How) are CIM approaches has been integrated into such programs?

## Methods

 Against this background, a SR was conducted, based on the PRISMA statement for SR (PRISMA-ScR) by Tricco and colleagues.^29,30^ A protocol was registered on 2023/07/30 at OSF (https://osf.io/xutc5/?view_only = 3bd56416ab524a5090eab4fac9062349).

 The SR review allows a broad overview about characteristics and key factors of WHP programs in long-term care facilities – along with an “*in depth examination” *of particular aspects of interest such as CIM and input from Experts.^29,31,32^ An SR method is ideal too “*identify, map, report, or discuss” *field concepts,^31^ such as the identification of characteristics and key elements of WHP programs in long-term care facilities that may impact the future practice and research, but where critical appraisal or risk of bias not necessarily needed.^32^

 To extend beyond the existing published knowledge, and gain insights related explicitly to the setting and target population in Germany, expert interviews were conducted with specialists in multimodal WHP programs in German long-term care facilities as a second database.^33^ The interviews aimed to specifically identify barriers and facilitating factors for the development and implementation of a multimodal WHP program in long-term care facilities on the part of practitioners. The content of the semi-structured question guideline for the interviews was developed from the results of the SR, with an orientation on an explanatory sequential mixed-method design.^28^ The SR expanded by this second data collection step that deepened the analyzed study results with experts’ experiences. This corresponds to the overall aim of an explanatory sequential mixed-methods design in which the second, qualitative dataset supports, explains and/or build upon the initial dataset.^28^ Unlike the original mixed methods approach, the first data set of this SR is not based on empirical data but on the findings of the SR. Applying the explanatory sequential mixed-methods design to the SR enables the expert interviews to be integrated into the findings, thereby extending them. The following section first explains the methodological steps for the SR and then for the expert interviews.

###  Methodological steps: Scoping review

 Publications eligibility criteria are specified according to the PCC (population, concept, and context) search process strategy.^31^ We included primary studies and study protocols that appeared in the last 15 years^[1]^ with focus on: professional caregivers, nurses, and other staff in long-term care facilities; multimodal WHP programs, composed of more than one single measure and published in English and German. All study designs were included. To avoid exclusion of publications that included, yet potentially expanded beyond long-term care facilities, no restriction on settings was initially applied to the search strategy. In the process, however, we excluded studies with no reference to long-term care settings.

 Articles screened which met the inclusion criteria were integrated and reviewed in the next step of the scoping review process.

 Publications were excluded that related to but were not focused on the multimodal WHP program. This was the case for publications in which the intervention reported on was not the multimodal WHP program itself, but only referred to certain elements, to methodological developments, or to the assessment of preconditions. Studies were further excluded in which a) the interventions did not have a multimodal character; b) nurses were not included as a target group; c) long-term care facilities were not addressed as a setting in the study.

 The search strategy took place in the databases Medline (PubMed), EMBASE (Ovid), and CINAHL (EBSCOhost) from February to April 2023. Boolean operators for the terms (workplace/ occupational/ corporate) health promotion/ service, and nurs*/caregiver* were used in a multi-stage, iterative process and adapted to the corresponding database. In a second step, an extended search was carried out by a second person (AS) to avoid overlooking relevant publications. In addition, manual reference list searching was conducted.

**Figure 1:**
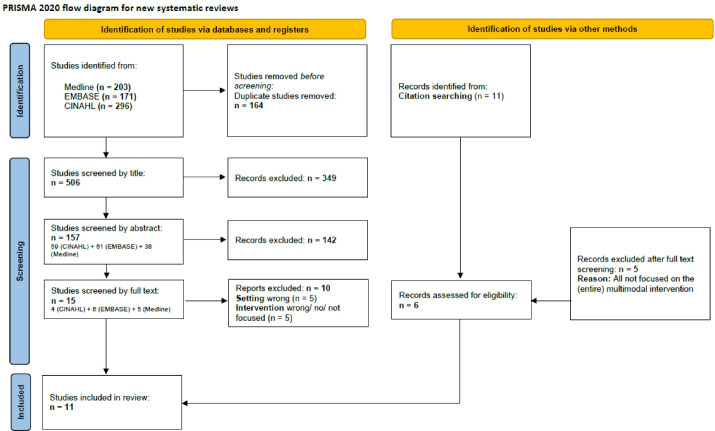
PRISMA flowchart^.^ Adapted version from:Page et al.^35^ For more information, visit: http://www.prisma-statement.org/

 Orientated on the methodological steps for SR by Macdonald and colleagues, the selection of relevant studies started with sorting the titles.^34^ All titles sorted as relevant were carried over to a next step, where the abstracts were reviewed. The remaining publications were subjected to a full text review. In addition, references of significant studies and review articles were screened to identify any further relevant studies as appropriate.The review process was undertaken by two independent researchers (JC, AS) and discussed regularly in a larger research team for consensual decision-making. The detailed selection of publications is shown in a PRISMA flowchart in Figure 1.^35^

 Further data management and analysis was performed with the software MAXQDA, version 2022.

 The included studies were analyzed through a systematic and descriptive process, starting with a full text reading of all included papers to generate a first broad overview over character and content. Parallel, the key characteristics (a) of the studies and (b) the of the interventions were summarized using a structured qualitative content analysis approach.^36^ First, a deductive category system using the research questions was established to systematically search the publications for the contents of interest in several steps: In a first summary, verbatim quotations from the publications were taken in order to reduce them to the essential statements. In a second and third read and analysis, higher categories of summaries were created. When appropriate inductive categories were developed from the material of interest, the category system was iteratively refined. This deductive/inductive approach allowed for a broader inclusion of content in the analysis not included beforehand in the deductive category system and is common for SR.^31^

###  Methodological steps: Expert interviews

 To include adult experts with a variety of different experiences in implementing WHP in long-term care facilities, a purposive sampling focused on maximum variation in terms of professional background was conducted.^37^ Contact with potential participants was set up with the help of gatekeepers. All experts gave written informed consent to participate. The interviews were conducted digitally via video conference, recorded on tape, and transcribed with the transcription software f4x. The transcripts were checked manually and then analyzed using the software MAXQDA 2022. Deductive categories developed from the results of the scoping review were used for a qualitative structured content analysis.^36^ The key findings of the expert interviews, according to challenges and facilitators for multimodal WHP, were synthesized with the correspondent results of the review to include knowledge and experience gained in practice.

## Results

 The search yielded 670 hits, 506 hits with duplicates excluded, in the first search run. After reviewing title and abstract, 15 publications remained for full-text review. From these 10 publications were excluded due to inappropriate setting^38-42^ and/ or intervention (no intervention,^43^ focus of the article is not on the intervention,^44-46^ lack of multimodal components/ intervention not specified^47^). Finally, 5 publications were included in the SR. Another 6 publications were added from the manual review of references (see Figure 1).

 The expert interviews were conducted with a total of 8 experts from the field of WHP in the care sector. The duration of the interviews ranged between 36 and 62 minutes. All the experts had expertise and experiences with WHP in care facilities from different point of views. The professional backgrounds are shown in Table 1.

**Table 1:** Expert interviews professional backgrounds

**Pseudonym**	**Professional background**
Ex_01	Health promotion in the care sector (service provider)/ Nurse
Ex_02	Personnel management geriatric care facility
Ex_03	Management and organizational consulting (focus on health economy, prevention, and health promotion)
Ex_04	Health management (health insurance)
Ex_05	General manager in a corporate group of care facilities / Health management/ Occupational Therapist
Ex_06	Managing director of care facilities/ Nurse
Ex_08	Head of care facility
Ex_09	Project management at a service provider for WHP

###  Part 1 – Study characteristics

 The final 11 studies contain a total of 6 interventions and are clustered in 6 intervention groups (IG), listed in column 1 of Table 2. Two of the SG include only one WHP intervention each (SG* 1*: ergonomic, strength, and resisting training^48^; SG* 2*: integrated health program^49^).

**Table 2:** Study characteristics

**(IG) Author/s, (Country)**	**Intervention**	**Objective of the paper**	**Design/ Approach **	**Target group (n=Per Protocol (PP)), Setting**	**Outcomes,** **Measurement instruments**	**Key results**
(1) Otto et al^59^ (Germany)	Combination of a tailored ten-week ergonomics and a twelve-week strength and resisting training **Duration** 10 weeks Disciplinary context* Department of Human Movement Science	To investigate the effect of the intervention on lifting behavior, strength endurance, LBP, and functional impairment caused by back pain	Crossover single blinded RCT	**Target group** Nurses and nurse aides, after dropout rate of 38%: IG n = 20, CG n = 22 **Setting** 2 nursing homes in Germany	**Primary outcomes** Lifting performance (PILE-Test), strength endurance of the lumbar extensors (Biering-Sørensen-Test), subjective perception of LBP intensity (VAS) **Secondary outcomes** Functional impairment caused by back pain (ODI), adherence (documentation of reasons for dropout and self-developed questionnaire)	**Primary outcomes** Improved lifting performance and reduced LBP of the IG compared to the CG after ergonomics training **Secondary outcomes** No differences between IG and CG for the Biering-Sørensen-Test and the ODI. Positive adherence rates in the IG were observed. The results of the questionnaire showed an overall positive evaluation of the program by the participants
(2) Tveito and Eriksen,^49^ (Norway)	Integrated health program, consisting of physical exercise, stress management training, health information and examination of participants’ workplace **Duration** 9months Disciplinary context* Faculty of Psychology	To assess if an Integrated Health Program reduce sick leave, subjective health complaints, and increase coping of nurses	RCT – pilot study	**Target group** Nursing personnel (nursing auxiliaries, nurses, assistants without formal education, other helping staff), after dropout rate of 27.5%: IG n = 12, CG n = 17 **Setting** 1 nursing home for older people in Norway	**Primary outcomes** Sick leave (documentation, supplied by the nursing home), subjective Health Complaints (Subjective, Health Complaints Inventory - SHC) **Secondary outcomes** Coping (Instrumental Mastery Oriented Coping Factor from the CODE), psychologic demands (5 questions from the Swedish version of the Psychological Demands dimension from the Demand/Control Model), control (6 items from the short Swedish version of the Decision Latitude dimension from the Demand/Control Model), health related quality of life (generic health status inventory SF-36), subjective effects of the intervention (7 statements to score on a 5-point scale), job stress (measurement not mentioned), effort reward imbalance (measurement not mentioned)	**Primary outcomes** There were no statistically significant effects on sick leave and subjective health complaints (exception: fewer neck complaints were reported in the IG compared to the CG) **Secondary outcomes** There were no statistically significant effects on health-related quality of life, coping, job stress, effort reward imbalance, demands, and control.
(3a) Balk-Møller et al^50^ (Denmark)	**(3a+b)** ^50,51^ Web- and App-Based Workplace Intervention to Promote Healthy Lifestyle and Weight Loss (SoSu-life tool) in combination with a social feature **Duration** 38 weeks Disciplinary context* Department of Nutrition, Exercise and Sports	**(3a)** ^50^ To investigate the effect of the SoSu-life tool in combination with a social feature on changes in body weight **(3b)**^51^ To investigate the motivation behind taking part in the interventions, and to explore the practical and social experiences of the participants with the tool	**(3a)** ^50^ RCT **(3b)**^51^ Qualitative Design	**(3a)** ^50^ **Target group** Employees from nursing homes, after dropout rate of 52%: IG n = 152, CG n = 117 **Setting** 20 nursing homes for elderly people in Denmark **(3b)**^51^ **Target group** Employees from nursing homes of the IG (active users and non-users of the tool), n = 26 **Setting** Nursing homes for elderly people in Denmark	**(3a)** ^50^ **Primary outcomes** Changes in body weight (digital electronic scale: Tanita WB 100MA/WB-110MA III) **Secondary outcomes** Changes in body fat percentage (handheld body composition monitor (Omron BF306)), waist circumference (measuring tape), blood pressure (digital blood pressure manometer (Kivex, Automatic Blood Pressure Monitor, Model UA-787 Plus), total cholesterol (finger-prick blood samples (Accutrend Plus) **(3b)**^51^ Exploration of the participants’ experiences with the tool, via Qualitative Interviews (n = 24) Focus Groups (n = 2 à 7 participants each)	**(3a)** ^50^ **Primary outcomes after 38 weeks** IG had lost -1.01 kg more body weight than the CG **Secondary outcomes after 38 weeks** IG had lost -0,8% more body fat percentage, and a larger decrease in waist circumference of -1.79 cm thang the CG **(3b)**^51^ **Motivation** by getting help and information by getting the physiological data on the bodily measures by the social part of being part of a team **Use of the SoSu-life tool** The usage of the tool was highest when the team competitions took place, which was in the first 16 weeks of the intervention period. Around day 40 the gradual decrease in use started. Roughly 66 % of the participants did not use the tool after its introduction at all. The social features met greater interest and got used more than modules about diet & exercise. **Experiences ** The SoSu-life tool motivated employees to participate in social activities at work and seemed to stimulate more social interaction around healthy lifestyle issues and habits.
(3b) Balk-Møller et al^51^ (Denmark)						
(4a) Gregersen et al^53^ (Germany)	**(4a+b)** ^52,53^ Qualification Program for Prevention of Psychic Stresses through Human Resources Development **Duration** 3 months **Disciplinary context** **(4a)**^53^ Department for Prevention and Rehabilitation (Berufsgenossenschaft für Gesundheitsdienst und Wohlfahrtspflege, BGW) **(4b)**^52^ Faculty of Social and Behavioural Sciences, Working Group Psychogeriatrics	**(4a)** ^53^ To increase in-house health through staff development (methodical, social and selfcare competences) **(4b)**^52^ To describe how the findings from the pilot phase have been integrated into the program and what modifications have been carried out To evaluate the quality of the training courses for the multipliers and of the implementation of the program by the multipliers	**(4a)** ^53^ Quasi-experimental design **(4b)**^52^ Evaluation of the experiences of the multipliers in the implementation the program via questionnaires and interviews	**(4a)** ^53^ **Target group** Employees from nursing homes, after dropout rate of 11%: IG n = 88, after dropout rate of 56%:CG n = 56 **Setting** IG = 11 nursing homes for elderly people in Mannheim/ Germany, CG = 10 nursing homes for elderly people in Heidelberg **(4b)**^52^ Multipliers (trained in 9 groups of 10-12 participants) from 16 model facilities	**(4a)** ^53^ Workplace related stress and resources Activity and analysis procedure for the hospital in the self-observation procedure (Tätigkeits- und Analyseverfahren für das Krankenhaus im Selbstbeobachtungsverfahren, TAA-KH-S)/ Procedure for self-monitoring of the operating climate (Verfahren zur Eigenkontrolle des Betriebsklimas, BKF) Professional competence in nursing care Questionnaire for the assessment of professional competencies (Fragebogen zur Erfassung beruflicher Handlungskompetenzen, FPK-A) Workplace related stress in geriatric care Scale for workload assessment Mental stress Questionnaire on stress in human services (Fragebogen zur Beanspruchung in Humandienstleistungen, FBH]/ General Health Questionnaire) Other health related aspects Standardized instruments for the evaluation of inpatient geriatric care facilities (Standardisiertes Instrumentarium zur Evaluation von Einrichtungen der stationären Altenhilfe, SIESTA)/ Biogramm/ Social Interview Schedule/ Competence and control examinations (Fragebogen zur Kompetenz- und Kontrollüberzeugungen, FKK) **(4b)**^52^ Description of the evaluation results of the pilot-study (4a) Experiences with and results of the program implemented by the multipliers in the 16 model facilities, through questionnaires and interviews (not further specified).	**(4a)** ^53^ The program could successfully develop the caregivers’ professional competences for reducing their job stress. The participants’ self-care skills improved, their occupational stress was reduced and the climate with the residence was enhanced. Nevertheless, statistically significant effects where only seen in the change of the climate with the residents (IG compared to CG). **(4b)**^52^ Based on the results of the pilot-study **(4a)**, a 5-step plan was developed to guide the implementation of the program in additional facilities for elder care: steering committee needs assessment and educational planning implementation transfer control success control The didactic and content density of the trainings for multipliers was high.The program could be successfully implemented in the facilities Some general conditions for a sustainable success of the program were identified, such as having enough time for the process of implementation and having the management involved.
(4b) Zimber et al^52^ (Germany)						
(5a) Kotejoshyer et al^54^ (USA)	**(5a+b)** ^54,55^ OSH/ (HP) program to facilitate employee participation (in examining and improving the physical, organizational, and psychosocial conditions at work that might impact health and well-being) **Duration** ~3-4 years Disciplinary context* (5a)^54^ Center for the Promotion of Health in the New England Workplace (CPHNEW) **(5b)**^55^ Department of Work Environment & CPHNEW	**(5a)** ^54^ To assess process fidelity in the intervention sites, extent of OSH/HP integration, health impact, and sustainability of the program **(5b)**^55^ To evaluate facilitators and barriers of the program after 3-year implementation	**(5a)** ^54^ Mixed-method evaluation design (convergent parallel strategy) **(5b)**^55^ Exploratory qualitative design	**(5a)** ^54^ **Target group** Employees IG + CG SurveyBaseline/Follow-up IG n = 360/331, CG n = 285/318 Interviews n = 47 Focus groups 3 with PIP team members, 3 with employees engaged in wellness at NPHP sites, 8 with employees at PIP & NPHP sites **Setting **6 nursing homes (IG) **(5b**)^55^ **Target group** Employees IG n = 58 in 8 focus groups/ n = 11 for individual interviews/ n = 13 interviews with the top management **Setting **3 nursing homes (CG)	**(5a)** ^54^ Process, integration, impact, sustainability of the program via surveys (baseline survey about the HP program activities in all 6 centers/ self-administered questionnaire about the workers chronic disease history, health beliefs and behaviors, and perceptions of the work environment, parts of the JCQ, 4 items to conduct safety climate [2 items from Griffin and Neal and 2 items developed by the researchers] **(5a+b)**^54,55^ Researcher observations Focus groups with team members / other nursing home employees Interviews with individual team members and wellness champions / management administrators and directors of nursing / supervisors **(5a)**^54^ for all 6 nursing homes (IG and CG) **(5b)**^55^ only for 3 nursing homes (IG)	**(5a)** ^54^ In all three intervention facilities the concept of OSH/HP integration was successfully operationalized with diminishing process fidelity over time. The work environment and health climate (e.g., respect) slightly improved. Higher employee engagement and more attention to organizational issues were reported at follow-up in two intervention sites. Resources available to the teams, management support, and changing corporate priorities affected potential program sustainability. **(5b)**^55^ Facilitators and barriers were reported from both managers’ and employees’ perspectives, and were categorized as intrapersonal, interpersonal, institutional, and corporate level. Management support, financial resources, and release time for participation were identified as the three most important factors
(5b) Zhang et al^55^ (USA)						
(6a) Rasmussen et al^56^ (Denmark)	**(6a+b+c)** ^56-58^ Multi-faceted workplace intervention consisting of participatory ergonomics, physical training, and cognitive behavioral training to prevent LBP and its consequences **Duration** 12 weeks Disciplinary context* (6a+b+c)^56-58^ National Research Centre for the Working Environment, Denmark, Institute of Sports Science and Clinical Biomechanics, University of Southern Denmark, Denmark, and **(6c)**^57^ Department of Occupational Medicine, Holbæk Hospital, Denmark	**(6a)** ^56^ To describe the design of the stepped-wedge multi-faceted cluster-randomized study **(6b)**^58^ To test the effectiveness of the intervention for lower back pain **(6c)**^57^ To test whether the intervention was effective for physical capacity, work demands, maladaptive pain behaviors, SPo work ability and sickness absence due to LBP	**(6a)** ^56^ Study protocol **(6b+c)**^57,58^ Stepped-wedge cluster-randomized controlled design	**Target group** Mainly nurses’ aides in elderly care but also kitchen and cleaning personnel and service workers, after dropout rate of 24%: n = 586 **Setting** 4 districts in Denmark, nursing homes and home care	**Primary Outcomes (6b)** ^58^ LBP (measured as days, intensity (worst pain on a 0-10 numeric rank scale), and bothersomeness (days) by monthly text messages) **Secondary Outcomes (6c)**^57^ Physical exertion, occupational lifting, muscle strength, fear avoidance beliefs and support from management, work ability, and sickness absence due to LBP (self-rated by the participants and obtained by text messages)	**(6b)** ^58^ This study shows that the multi-faceted intervention could reduce LBP, pain intensity, and bothersomeness in eldercare workplaces (nursing homes and home care) in a group of workers mainly made up of nurses’ aides. **(6c)**^57^ The multi-faceted workplace intervention showed itself also effective for physical work demands and maladaptive pain behaviors, but not for work ability and sickness absence.
(6b) Rasmussen et al^58^ (Denmark)						
(6c) Rasmussen et al^57^ (Denmark)						

Abbreviations: LBP, low back pain; IG, intervention group; CG, control group; RCT, randomized controlled trial; ODI, Oswestry disability index; VAS, Visual analog scale; PILE, Progressive Isoinertial Lifting Evaluation; OSH, occupational safety and health; HP, health promotion; JCQ, Job Content Questionnaire.

 The multiple (sub)studies in the remaining 4 SG contain different perspectives, objectives and methodological approaches on the same WHP intervention (*SG 3*: SoSu-life tool^50,51^; *SG 4*: program for prevention of stress through human resources development^52,53^; *SG 5*: occupational health and safety/ health promotion program^54,55^; *SG 6*: multi-faceted workplace intervention^56-58^).

 The studies took place in 4 countries: Germany (*IG 1 and 4*), Norway (*IG 2*), Denmark (I*G 3 and 6*), and the USA (*IG 5*). They were conducted against various disciplinary backgrounds such as sport, movement, and nutrition (*IG 1, 3, and 6*), psychology (I*G 2*), prevention, rehabilitation, social and behavioral sciences (*IG 4*), health promotion and work environment (I*G 5, and 6*), and occupational medicine (*IG 6*).

 The characteristics, objectives, and focus of the studies vary widely. This becomes particularly apparent in the following points:

1. **Design**: 6 studies follow a quantitative design^49,50,53,57-59^ of which 5 studies are randomized controlled trials (RCTs), 3 have a qualitative approach,^51,52,55^ one study is a mixed-methods study,^54^ and one is a study protocol.^56^
2. **Objectives**: Most of the IG aim to reduce stress (*IG 2, 4*), strengthen physical and psychosocial abilities and resources (*IG 1, 2, 6*), or address health/ healthy lifestyle in general (*IG 3, 5*). One IG focuses on weight loss (*IG 3*), another on the facilitation of employee participation within the organization (*IG 5*).
3. **Duration**: The duration of the implementation process ranges from 10 weeks (*IG 1*) to 4 years (*IG 5*).
4. **Outcomes**: The primary outcomes of interest relate to low back pain,^58,59^ stress,^53^ body weight,^50^ sick leave, subjective health complaints,^49^ lifting performance and strength endurance.^59^
5. **Effects**: The results of the studies with quantitative approaches have been mostly positive with regard to the primary outcomes. For example, improved lifting performance,^59^ weight loss,^50^ stress reduction, improved staff-resident relationships,^53^ improvement of health climate, work environment, and employee engagement^54^, fewer neck complaints,^49^ and reduced lower back pain^58,59^ could be observed. However, not all results are statistically significant, e.g., Tveito and Eriksen point out that their WHP did not show statistically significant effects on sick leave and subjective health complaints.^49^ In contrast, subjective perceptions (measured with statements to score) of the participants showed positive effects of the WHP on health and well-being, in particular on physical fitness, muscle pain, stress management, and maintenance of health and work situation. Only one of the qualitative studies specifically refers to effects of the WHP and assumes that the SoSu-life tool motivates employees to participate in social activities at work and stimulates social interactions around healthy lifestyle issues and habits between the personnel.^51^ The other two qualitative studies focused on the evaluation of facilitators and barriers for the implementation of the tool, not on the perceived effects of the WHP.^52,55^

###  Part 2 – Multimodal WHP’s characteristics 

 The structures, contents, and specifics of the WHP programs are summarized in Table 3.

**Table 3:** Program characteristics

**Multimodal components of the program**	**Structure of the program**	**Structural integration within the settings’ conditions**	**Challenges of the program/ barriers **	**Potential of the program/ facilitators**
(1)^59^ Multicomponent exercises to prevent and reduce back pain 1. 10-week ergonomics training 2. 12-week strength and resisting training	1. 10-weeks ergonomics training •Duration (training): 1 session (S) a week for 10 weeks •Duration (S): 20-30 minutes/ •Participants: 6-8 participants per group •Content (S): S 1 = work organization, S 2-3 = standing & positioning, S 4-5 = working on the care bed, S 6-8 = transfer situations, S 9-10 = nursing aids and summary 2. 12-week strength and resisting training •Duration (training): 1 session a week for 12 weeks •Duration (S): 45-60 minutes •Participants: number of participants = not mentioned •Content (S): Each S includes 4 parts (P): P1 = mobilization and warm-up, P2 = coordination, P3 = circuit strength training, P4 = relaxation.	Level of participatory approach/ involvement of staff and setting: •Adaption of the program on the settings and working conditions •The intervention took place during working hours •Adaption of training intensity according to the participants’ level of fitness	Time pressure, lack of time, lack of motivation due to the training time during working hours	Positive adherence to the program •Nurses regularly participated in the intervention •Nurses accepted and tolerated the intervention The authors suggest that the participatory approach may have led to the positive adherence to the program Positive effects on lifting performance, ergonomic behavior, and LBP
(2)^49^ IHP 1. Physical exercise 2. Stress management training and health information 3. Examination of the participants workplace	1. Physical exercise •Duration (training): 3 times a week for 9 months •Duration (S): 1 hour •Participants: number of participants = not mentioned •Content (S): Body awareness, warm-up/aerobics/ergonomics, cool-down exercises, strength/stabilizing, stretching, relaxation 2. Stress management training and health information •Duration (training): 1 session a week for 15 weeks •Duration (S): 1 hour •Participants: number of participants = not mentioned •Content (S): Health and lifestyle information, stress management training, discussion •Workplace examination	Level of participatory approach/ involvement of staff and setting: •The intervention took place during working ours •The staff was granted leave from work to participate •It was ensured that the departments were left with sufficient staff	The IHP may have increased the workload of the CG High drop-out Increase of sick leave in IG and CG during the intervention	Positive perceptions of the effects on health and well-being (IG) •Physical fitness •Muscle pain •Stress management •Maintenance of health and work situation The IHP may be of use to increase satisfaction and well-being among employees Positive attitude of the IG and the CG towards the IHP
(3)^50,51^ Web- and App-Based Workplace Intervention (SoSu-life tool) 1. BCT 2. Individual feedback system 3. Social features	•Duration: 38 weeks •Participants: n = 355 •Content: The participants could choose between 7 pledges according to behavior change techniques (lose weight, eat healthier, improve physical fitness, improve physical strength, quit smoking, decrease the number of cigarettes smoked, maintain a healthy lifestyle). The program had various tools to help the user succeed with the pledges such as individual feedback system and social features.	Level of participatory approach/ involvement of staff and setting: •Individual program recommendations were based on results of health examination •Local health celebration events at the nursing homes •Participation was voluntary	Tool was perceived as technically too difficult, time-consuming, and not useful Reactions such as demotivation, aversion against healthy lifestyle, bad conscious as a result of the intervention Long-term effects were modest and associated with a relatively high dropout	Focus on social interactions and community elements were positively highlighted Incorporated social support through individual feedback system and social features SoSu-life tool maintains a weight loss over the intervention period
(4)^52,53^ Workplace Health Promotion through Human Resources Development Modular system with 10 qualification modules to foster the social, personal, and methodological competences of the target group	•Duration (training): 12 S over 12 weeks •Duration (S): 90 minutes •Participants: max. 12 participants per training group (14 training groups) •Content (S): Session 1-4 Dealing with “challenging” clients/ patients, Session 5-8 Professional self-perception, dealing with stress and personal problems, Session 9-12 Communication and Leadership	Level of participatory approach/ involvement of staff and setting: •Training and involvement of facilitators •Sessions were framed as In-house training •Needs assessment with the involvement of a steering committee composed of the facilities' staff •Composition of the program adapted to the specific needs of the respective care facilities staff (selection of relevant topics from a modular system)	Circumstances that were perceived as inhibiting •Program was reported as too dense in terms of content and time •transfer of what was learned to everyday practice •lack of staff and staff shortages due to program •inadequate information flow •lack of support from the management •lack of motivation •too large intervals between the sessions	Facilitating elements for the program’s success •the management accepted and supported the program •the participants had specific learning expectations and goals •the multipliers showed a high flexibility and willingness to cooperate •good communication culture, openness to change and trust in the employees in the facility •Creation of a safe space for the sessions and regular participation •Involvement and active participation of the target group in the program development and implementation •High engagement of the steering committee
(5)^54,55^ Workplace participatory occupational health/health promotion program PIP that integrates OSH with HP	•Duration (training): 5 years •Duration (S): PIP team members meeting every two weeks for about 1 hour •Participants: 10 participants per PIP team •Content (S): PIP teams were created to address integrated workplace HP and OSH concerns, adapted to their working and setting conditions, and the staffs’ needs. Traditional health promotion activities (e.g., nutrition, exercise) as well as work organization, psychological stress, ergonomics concerns and other occupational health and safety issues are included.	Level of participatory approach/ involvement of staff and setting: In the explicitly participatory approach of the PIP, the employees are actively engaged in problem identification, program design, implementation, and evaluation of the program	•Lack of communication •Peer pressure •Lack of participation •Top-down decision-making structures •Lack of financial support •Difficulty with time release •staffing shortages, time constraints, and clinical care responsibilities •sustainability suffered due to lack of resources and inconsistent management	3 factors that have been shown most important for success •Management support •Financial resources •Release time for employee participation •The following has proven to be supportive •Leadership development training with a focus on health protection and health promotion •Organizational support •Empowerment of front-line employees in decision making Budget for health and wellness activities •Employee awareness and engagement •participatory approach •In-house resources
(6)^56-58^ Multi-faceted workplace intervention to prevent LBP and its consequences 1. Participatory ergonomics 2. Physical training 3. CBT	1. Participatory ergonomics Duration (training): 5 sessions over 12 weeks •Duration (session): startup meeting = 1 hour, two workshops = each 3 hours, two follow-up meetings = each 1 hour •Participants: 5 to 7 participants per group •Content (session): prevention of physical exertion and pain •Physical training •Duration (training): 12 sessions over 12 weeks Duration (session): once a week for 1 hour •Participants: all participants •Content (session): body awareness, strength and coordination, physical activity •CBT •Duration (training): 2 sessions over 12 weeks •Duration (session): each session 3 hours •Participants: all participants •Content (session): Thematic focus on understanding, experiences, and anticipation of pain, ability to function and have a good quality of life despite pain	Level of participatory approach/ involvement of staff and setting: •Intervention was scheduled in the working time of the participants •Focus on participant and organizational involvement •Organizational commitment •Integration of the program to the organizational health system •Supervisors were trained to support the intervention •An employee ambassador has been appointed in each team to motivate the colleagues	•Low participation rate •The integration of the components 1., 2., and 3. only allows for evaluation of the effects of the entire intervention •The multifaceted intervention requires more resources than single components	Facilitating elements for the program’s success External resources for the participating workplace •Strong commitment from the management and the organization •The intervention was delivered by local trained therapists with potential of adding local knowledge and sustainability •The multifaceted intervention shows high potential to maintain the participants’ function at work, work ability, productivity, and quality of life through reduction of LBP days, pain intensity and a reduction in bothersomeness •The multifaceted components meet different needs

Abbreviations: LBP, low back pain; IG, intervention group; CG, control group; IHP, Integrated Health Program; BCT, Behavior-change techniques; PIP, Participatory intervention program; OSH, occupational safety and health; HP, health promotion; CBT, Cognitive behavioral training

 In line with the different aims and outcomes of the studies, the multimodal components of the described 6 individual WHP interventions, their content and composition also varied considerably.

 Regardless of the specific content of the WHP programs, several underlying distinguishing features were identified: One distinction concerns the temporal closure of the programs. While some programs can be seen as self-contained units that end after each pass (IG* 1, 2, 4, 6*), an app based WHP could continue to be used by the staff after the study was completed (*IG 3*). Another program (*IG 5*) has developed health-promoting structures in the participating facilities together with the employees that are aimed at long-term change.

 This leads to another important distinction between the programs, which is the level of employee involvement in the development and implementation of the WHP programs. Three approaches can be distinguished: (a) the entire program is developed and implemented together with the staff of the facilities according to their needs, expectations and preferences and the circumstances of the setting (*IG 5*); (b) an already existent program(-frame) is adapted to the needs and preferences of the target group and the circumstances of the setting under participation of the staff (*IG 1, 4, 6*); (c) an already finalized program is implemented without participatory involvement of the staff concerning content-decisions and structure (*IG 2, 3)*.

 In one case the delivery of the WHP program took place web- and app-based WHP (*IG 3*). The other WHP programs provided their content/exercises in several on-site presence-training sessions or modules (*IG 1, 2, 4, 6*).

 Common to all interventions is the multimodal composition of the program, which consists of several individual measures with different contents, orientations, and structures. Some studies explicitly justify their choice of a multimodal WHP structure more on a meta-level than related to the specific content of the program. The explanations refer to different initial hypotheses: High requirements and strains of the employees call for the most versatile approach possible to WHP, which is (more likely to be) covered by opting for multimodal elements (*IG 1*); A multimodal approach is considered to be more effective in motivating those involved (*IG 2*); Employees need a bundle of competences/ key-qualifications to effectively prevent stress (*IG 4*); Multimodal components enhance the potential that very different needs of the participants can be successfully addressed (*IG 6*). *IG 5*, however, gives a more content-specific explanation for the multimodal character of the intervention: The aim here is to enhance the effectiveness of WHP by combine it with occupational safety and health approaches. Only *IG 3* do not explicitly justify the motivation for choosing a multimodal versus a singular intervention.

 CIM approaches can be seen in all programs, at least to some extent. Yet these are mostly limited to mind-body approaches such as mindfulness training, stress management via for example yoga techniques and massages, and strategies for increasing body awareness (*IG 1, 2, 4, 5, 6*). Topics such as healthy nutrition (*IG 3*) also overlap with CIM approaches. However, none of these approaches are explicitly embedded within a CIM framework, and no program shows an overall orientation to CIM approaches. In contrast, some of the interviewed experts described their support for the use of CIM measures in WHP programs, stating they would be potentially particularly beneficial for WHP (Ex_04, Ex_05, Ex_01), declaring their own approach as open to CIM desired by staff (Ex_02, Ex_08, Ex_06) and/ or are already implemented CIM approaches as one part of multimodal WHP programs (Ex_09).

###  Part 3 – Facilitators and challenges of the WHP: Integrated results from expert interviews and scoping review

 Although the differences in the interventions seem to be the salient common attributes they share, insights about general facilitation and barriers to a sustainable implementation of multimodal WHP programs can be identified and summarized from both the studies and the expert interviews.

####  Challenges

 Overall, the issue of drop out and decreasing participation rate is cited as a major challenge for most of the included studies (*IG 2, 3, 5, 6*). This despite the fact that all studies preventively implemented strategies in their program to encourage motivation and participation. Strategies included: Interventions – or parts of the intervention (*IG 2*) – conducted during working hours (*IG 1, 3, 6*). One program conducted an in-house-training (*IG 4*) while another gave time off of work for participation (*IG 5*). In the app-based intervention, participation was also encouraged through social interaction elements such as contests, prizes, and celebrations (*IG 3*). Two studies described the dropout as relatively low (*IG 1, 4*). The reasons for the high vs. low dropout/ participation rates could probably be attributed to the overall challenges or barriers and facilitators of the intervention as perceived by the participants. The challenges, extracted from the publications, can be grouped into different categories, summarized in Table 4.

**Table 4:** Challenging parts of the Workplace Health Promotion (WHP)

**Categories ** **Reasons for challenges**	**Subcategories ** **Challenging themes**
Structural reasons	a)Lack of Resources •Time related •Time consuming set up (IG* 3*) of the intervention during working hours can result in •time pressure (IG* 1, 5*) •lack of time because of intervention during working hours (*IG 1, 5*) •staff shortage (*IG 4, 5*) •higher workload for those not taking part at the intervention (*IG 2, 4*) •peer pressure because of the higher workload for colleagues (*IG 5*) •Finance related •lack of in-house financial support (*IG 5*) •lack of corporate fundings (*IG 5*) b)Shift working structures •Limited participation through shift work (*IG 3*) •c)Hierarchical structures •Top-down decision-making structures (*IG 5*) •Inconsistent management (*IG 5*)
Working-atmosphere related reasons	a)Communication •Insufficient information flow (*IG 4*) •Lack of communication between all parties involved (*IG 5*) b)Support system •Lack of support from management (*IG 4*)
Intervention related reasons	a)Technic •App-based intervention might be a barrier for participants without experiences with smartphones (*IG 3*) •Technically too difficult (*IG 3*) b)Content •Too dense and overloading (*IG 4*) and not useful (*IG 3*) •Theory was challenging to transfer into practice (*IG 4*) c)Time •Time for the intervention was perceived too short for the content (*IG 4*) •Time between the sessions of the intervention were perceived too long (*IG 4*)
Intrinsic reasons	a)Motivation •lack of motivation (*IG 1, 4*) •demotivation due to focus on health (*IG 3*) b)Difficult emotional reactions bad conscious because goals are not achieved (*IG 3*) •peer pressure due to the increased workload for colleagues because of their own participation in the intervention (*IG 5*) •frustration with decision-making process and clinical responsibilities (*IG 5*)
Unknown reasons	Increase of sick leave during intervention (*IG 2*)

 With regard to the sustainability of the WHP programs, one study (*IG 4*) assumes that the selected intervention period of 12 weeks was not sufficient to achieve sustainable effects. Whereas the Participatory Intervention Program (*IG 5*) identified lack of resources and inconsistent management as the major barriers to a sustainable program implementation. The experts’ experience confirms most of the challenging aspects mentioned. In particular, the experts focused on time-related challenges (*Ex_05, 09, 04, 01, 03, 02, 08*), lack of support among management (*Ex_09, 03, 08*), and lack of financial support (*Ex_04, Ex_03*).

####  Facilitators

 Supporting elements for a sustainable implementation of multimodal WHP programs can be located primarily in three areas: Support by the management, good communication culture, and participatory approach.

 For support by the management both, studies (*IG 4, 5, 6*) and most of the expert interviews (*Ex_09, 04, 01, 03, 02, 08, 06*) strongly emphasize how relevant a positive, open, and supportive attitude towards a WHP project among care facility management is, especially when it comes to the acceptance of the program as the following expert quote highlights:

*“In this respect, they [the managers, JC] are very important allies. And the managers are also the only ones who are in a position to ensure permanence and consistency, to ensure sustainability. In this respect, they are the decisive players in quotation marks [... and] very important allies.” *(Ex_04).

 Not only is the relevance of openness for change and flexibility by the management towards health promotion highlighted (IG 4, Ex_09), but also from other involved stakeholders due to flexible adaptation of ideas to the needs of the target group, in particular the specific target group of nurses:

*“So, you need […] to look at it really closely, who is in front of me, how do I approach this person? And it has to be individual. So that’s. That is simply totally important in nursing. We can just take off with a standard program. It doesn’t work. It won’t work.” *(Ex_09)

 Flexible adaptation, coupled with good communication and regular dialogue within the facilities, it was listed as a benefit by both the publications (*IG 4, *implicitly also* IG 5, 6*) and mentioned by experts (*Ex_03, 05, 04, 02, 08, 06*). The relevance attributed to good communication between management and employees is exemplified by a working relationship that encourages dialogue:

*“If you have a managing director who rushes around, doesn’t talk to anyone [...] and then disappears into his room and is never to be seen, then you don’t need to do a WHP. It won’t work. Yes, so communication has always been the key for me in all areas. Yes, if you don’t have a relationship with your employees, if you can’t talk to them in three words, then any WHP is doomed from the outset.” *(Ex_03)

 Good, appropriate communication between those who implement a WHP in the institution (e.g., service provider, research group) and the staff is further emphasized in that communication with the participants, particularly nurses, should definitely avoid creating pressure and stress: *“So, in nursing it just can’t cause more pressure, because they have much too much of it during their regular workday.”* (Ex_09)

 The potential of a participatory approach to facilitate positive adherence, participation, and generally for the success of the WHP was highlighted by some studies (*IG 1, 4, 5*), as a participatory approach allows the tailoring to the setting’s specific context and individual needs of the employees into the WHP. The relevance of including specifics of the setting and needs of the employees is also mentioned by some of the experts, e. g. in form of a participatory approach (*Ex_05, 03, 08*), sometimes in addition to a needs assessment as background for WHP development, which was emphasized by all experts. In some studies (IG 4, 5, 6), the training and deploying of peer facilitators carried out as one part of a participatory approach to define and delegate responsibilities for the WHP. This is one approach to improve sustainability and discussed also by experts (E*x_05, Ex_04, Ex_03*).

*“In every facility [we train] a so-called health facilitator […] that carries on and carries out a bit what we’ve come up with from above into in the individual facility. Because that is an important point in my view. If no one feels responsible, a person can suggest and initiate all they want, but in the end it won’t be implemented.” *(Ex_05)

 Some experts consider the motivation and enthusiasm of the facilitators (*Ex_05, Ex_01*) for the project and the personal engagement of persons involved (*Ex_04, Ex_03*) to even be a key success factor for the acceptance of WHP by employees. According to *Ex_01*, one means of stimulating motivation and enthusiasm is self-experience in the measures that are to be part of the WHP:

*“And that is why we also, if at all possible, try to get the people excited […] Yes, and also the personal experience is a very important part […], the most important of all for the whole thing.” *(Ex_01)

 The facilitating factors for the success of WHP, taken from the studies and the experiences of the experts can be summarized and synthesized as key conditions for the development and implementation of a sustainable multimodal WHP program for care facilities in detail in Table 5.

## Discussion

 Eleven publications were included in the SR on 6 multimodal WHP interventions (*IG 1-6*). To further broaden and deepen the results of the SR, 8 interviews with experts from the field (see Table 1) were conducted.

 The findings highlight key challenges to the implementation of multimodal WHP programs. The obstacles primarily stem from structural challenges, including constraints in time, finances, and hierarchical complexities, as well as team dynamics characterized by a lack of support, communication issues, and low motivation. Conversely, the efficacy of the programs is positively influenced by effective communication, management support, and participatory engagement from the target group.

 The results obtained from the review and expert interviews were consolidated into an overview with essential preconditions for the development and implementation of a multimodal WHP program in long-term care facilities, concerning structural requirements, attitude/atmosphere, and workplace culture (Table 5).

 Notably, despite the frequent adoption of CIM approaches in the WHP interventions and the experts’ explicit emphasis on the potential of such approaches in WHP programs, it is noteworthy that the CIM discourse is not explicitly focused on in the literature, nor are there published multimodal WHP programs based on CIM approaches.

###  Variety of multimodal WHP programs 

 Although there are general guidelines for the development and implementation of WHP, for example in the form of declarations or checklists, there are no standardized rules for the design of health-promoting programs in long-term care facilities with regard to specific content, structures and outcomes.^17,46^ This is clearly reflected in the results of this SR: The WHP programs differ greatly regarding content, the outcomes of interest, the individual elements that are integrated into the interventions, the structure in which the interventions were implemented, and the degree of participatory approach. One explanation for this is the relevance of adapting a WHP to the specific needs and resources of the target group as well as to the conditions of the setting. This relevance is emphasized by both included studies and experts. A thorough needs assessment is therefore considered a basic precondition for WHP programs.^46^ Furthermore, one way of incorporating diverse needs, wishes, and work life challenges (*IG 1, 5*) and increase the motivation (*IG 2*) of employees in a WHP is to implement a multimodal approach that integrates various measures. Arguments for a multimodal approach are also found in expert interviews and in other sources.^22,23^

 In addition, the discussion about WHP does not seem to be specifically dominated by particular disciplines within the included publications but is of interest to different fields and contexts. This can be seen in what was underscored by publications, including: the focus on the health-promoting potential of (health-)education (*IG 4*) that addresses a health literacy discourse and its potential for health promotion.^60^ Another study focused on how health-promoting structures in particular were developed by the staff itself (*IG 5*) and thus referencing factors of self-determination, autonomy and strongly adapted WHP. It is also notable that despite recent, rapid technological developments, only one intervention of the included studies described an entirely web- and app-based WHP program (IG 3). Attention was drawn to the ambivalence of needing to use technology: some participants in the study rejected the program due to excessive demands when using the provided app. The challenges and opportunities associated with digital health tools that support WHP programs should be explored in future WHP studies.

 Despite the differences of the WHP programs, there are common factors and barriers for a successful and sustainable implementation of a multimodal WHP program that are found in many interventions emphasized by the projects/programs and the experts. The barriers include above all structural challenges such as time and financial resources, shift work, and hierarchies, and atmosphere-related challenges within in the team such as no support from management, poor communication structures, and low motivation. In turn, a good communication culture, motivation and support from management, as well as participatory involvement in the intervention development and implementation, appear to encourage the development and sustainability of the implementation of a multimodal WHP program and should be considered in future WHP interventions, always adapted to the specifics of setting and staff (see in more detail: Table 5).

**Table 5:** Key conditions for Workplace Health Promotion (WHP)

**Categories**	**Subcategories **
Structural conditions	1. Ressources: •Use and integration of in-house resources possible and encouraged (e.g., premises, equipment, staff expertise) 2. Willingness to accept new structures: •Willingness of management to provide time resources for staff and to develop structures to avoid staff shortages due to the participation in WHP •Openness and motivation of management and staff for healthy change and transformation within the setting •Implementation of health teams from within the workforce possible and encouraged •Willingness of the management to embed the participatory involvement of staff in the WHP into the structure of the organization •Willingness of management and all those involved to establish a steering committee made up of different functional, hierarchical and interest groups in the institution
Attitude/ Atmosphere	1. Management: •High motivation, commitment and openness towards the development and implementation of WHP within the facility •Willingness to commit time and resources to help develop and implement the WPH program •Mindset that staff needs and wishes must be included in the WHP program •Management is supportive of the project's participatory approach and is willing to facilitate its implementation •Management sees itself as part of the overall staff and as a target group for the WHP program •Management is convinced of the potential of CIM approaches in WHP •Openness to and support of motivational events and campaigns 2. Employees: •High motivation to actively participate in the development and implementation of a WHP program for the facility •(Part of the) team is convinced of the potential of WHP program
Workplace culture	1. Communication: Good communication culture within the facility 2. Involvement & participation of staff in decision-making: Wishes, needs and concerns of staff are heard and dealt with by the management 3. Trust: Trusting relationship between staff and management

###  Effects of multimodal WHP programs and methodological challenges of capturing them

 The results of the included studies indicate that the various approaches do have some effect. Despite the fact that these possible effects could not be all captured by statistical methods, the experiences and perceptions of the participants demonstrate the influence, as in the case of *IG 2*. While the qualitative statements emphasize positive effects, which might have a great impact on individuals’ well-being and subjective feelings of health, some of these cannot be demonstrated in a statistically significant way with quantitative data alone.^52^ This observation is consistent with the understanding that complex interventions are challenging to evaluate adequately in all relevant facets with only one methodological approach.^61,62^

 The same applies to CIM interventions, which are often multimodal per se and are considered complex interventions^63,64^ with a holistic approach that require whole systems research methods.^65-67^ A multimodal, CIM-based WHP program therefore requires multifaceted perspectives in order to assess the effects of the intervention as comprehensively as possible. This highlights the relevance of an adapted multi-method approach for the evaluation of multimodal, CIM-based WHP programs. Not least in order to be able to adequately identify the barriers and facilitators associated with the corresponding interventions. The relevance of qualitative methods in this field of research to assess the needs, experiences and opinions of the target group should be emphasized at this point. Including the views of the target group is essential for sustainable intervention development and implementation.

###  Challenges and facilitators of multimodal WHP programs 

 The studies and expert interviews revealed some challenges and facilitators that were repeatedly mentioned and/or particularly emphasized regarding the development and implementation of multimodal WHP programs.

 One challenge repeatedly mentioned in the included publications and by the interviewed experts is related to the resource of time, consistent with research found on WHP program implementations for care professionals/ nurses.^43^ The chronic deficit of nursing staff, and the work demands placed on nursing staff to attend to the health of others, hinder time devoted to their own health.^3^ The participation rate in WHP programs is therefore often low,^68^ the drop-out rate high^49^ and motivation difficult to build up and maintain.^43^ Various solutions are conceivable to meet this challenge, as the results of the scoping review and the expert interviews indicate: Similar to what has been stated elsewhere^43,44,45^ the results show that management support is one of the most relevant prerequisites for the success of a WHP program, and thus also the overall motivation of management and the target group with respect to WHP. In addition, a good general communication culture within the facility and the involvement of employees in the development and implementation of a multimodal WHP program appear to be particularly relevant factors. Arguments for a participatory approach under consideration of multiple perspectives and the value of communication within this approach are highlighted elsewhere.^43,69^ Involvement of active participation on WHP is even considered as an essential part of a successful WHP.^17^ This is in line with Otto et al, which assume that the participatory approach may have led to a good adherence to their multimodal WHP program.^59^

 Because WHP interventions almost always contain interventions classified as CIM, they would benefit from stating so explicitly. The opportunities of a WHP based entirely on the principles of CIM, as described in the introduction and confirmed in some of the expert interviews, have therefore not yet been focused as means of choice in research. The same applies to specific challenges associated with the implementation of a multimodal program that is explicitly and completely based on CIM approaches. This reveals a gap that can and should be explicitly addressed in future project developments.

## Strengths and limitations

 The integration of a SR with empirical data from expert interviews in the spirit of an adapted sequential mixed-methods explanatory design^28^ is a methodological innovation that does not follow a predetermined structure. Instead, the steps were presented as transparently as possible in order to ensure traceability. The synthesis of results from a review with empirical data appears to be extremely profitable and should be further explored and methodologically differentiated in future approaches.

 Some limitations of this scoping review should be mentioned in order to increase rigor: Grey literature was not included for reasons of resource practicability. In order to incorporate expertise and experience from the practical side and in relation to specific circumstances in Germany, expert interviews were conducted instead, which support and expand on the results of the scoping review. We focused on the experience of experts in Germany because this scoping review is intended to serve as preparation for the development, implementation and evaluation of a multimodal WHP in long-term care facilities in Germany. The experiences of experts from other countries may differ strongly and offer deeper insights into challenges and opportunities of multimodal WHP programs in long-term care facilities. We recommend that future empirical research projects within this field explicitly adopt and focus on cross-cultural and international perspectives.

 With regard to the search strategy for the inclusion of relevant publications, a limitation to a total of 3 databases (PubMed, EMBASE, CINAHL) was compromised for resource reasons. It is possible that further publications could have been found in other databases that would have met the inclusion criteria. However, the most relevant databases for the topic were selected and, in addition, a comprehensive source search was carried out to keep this limitation as low as possible.

## Conclusion

 The findings of this SR are in line with the “Luxembourg declaration on WHP in the European union”.^17^ Specifically, this refers to the inclusion of a participatory approach, awareness of the relevance of health promotion structures in organizations, WHP development through a structured project management process, and the integration of both individually oriented and environmentally orientated measures from different areas ( = participatory approach and multimodal structure). The results of the SR and the expert interviews were synthesized into an overview of relevant key conditions for the development and implementation of a sustainable multimodal WHP program (Table 5) in long-term care facilities. The following key points were considered and differentiated: Structural preconditions, attitude/atmosphere, workplace culture. In particular, the willingness and commitment of the management was highlighted as a key factor for sustainable success of a WHP program. That includes for example a high motivation, commitment and openness towards the development and implementation of WHP within the facility, and a general willingness to commit time and resources to help develop and implement the program (see in detail Table 5). The overall results reveal that approaches from the spectrum of CIM occur in all included interventions. However, none of the publications refers explicitly to the CIM discourse, which is stated as highly relevant in WHP also by interviewed experts. The potential of a multimodal WHP program that makes exclusive use of CIM strategies has not yet been investigated for the setting. This should be subject to further research and implementation practice. Furthermore, the challenge of the appropriate evaluation of complex interventions, such as multimodal WHP programs are, is evident. A multi-method approach is therefore recommended as the standard for further research in this area. Finally, we would like to encourage future empirical studies on multimodal, CIM-based WHP programs to adopt and compare cross-cultural and international perspectives. This could significantly expand the knowledge in this relevant area of interest.

## Acknowledgements

 We would like to warmly thank the interviewed experts for their valuable support of our SR.

## Ethical Approval

 The empirical part of the SR was approved by the ethical committee of Charité – Universitätsmedizin Berlin (EA2_204_22, 10/01/2023).

## Endnotes

^[1]^ The time restriction is based on the intensified discussion about the relevance of WHP after the endorsement of the *Global Plan of Action on Workers Health (GPA) *by the WHO.
